# Traumatic rhabdomyolysis: rare but morbid, potentially lethal, and inconsistently monitored

**DOI:** 10.1007/s00068-023-02420-8

**Published:** 2024-03-27

**Authors:** Thomas Giles, Kate King, Simone Meakes, Natasha Weaver, Zsolt J. Balogh

**Affiliations:** 1 Discipline of Surgery, School of Medicine and Public Health, University of Newcastle, Newcastle, NSW Australia; 2 Department of Traumatology, John Hunter Hospital, Newcastle, NSW Australia; 3 Injury and Trauma Research Program, Hunter Medical Research Institute, Newcastle, NSW Australia

**Keywords:** Rhabdomyolysis, Traumatic rhabdomyolysis, Acute kidney injury, Polytrauma

## Abstract

**Purpose:**

Although traumatic rhabdomyolysis (TR) is shown to be associated with acute kidney injury (AKI), there are no large prospective epidemiological studies, interventional trials, official guidelines outlining the appropriate investigation, monitoring, and treatment on this poorly understood condition. We aimed to establish the contemporary epidemiology and describe current practices for TR to power future higher quality studies. We hypothesised that investigation and monitoring occur in an ad hoc fashion.

**Material and methods:**

We conducted a 1-year retrospective cohort study of all patients > 16 years of age, with an ISS > 12 and, admitted to a level 1 trauma centre. Demographics, initial vital signs, admission laboratory values, and daily creatinine kinase (CK) values were collected. The primary outcome was TR (defined by CK > 5000 IU), secondary outcomes included AKI (KDIGO criteria), mortality, multiple organ failure, length of stay, and need for renal replacement therapy (RRT).

**Results:**

586 patients met inclusion criteria and 15 patients (2.56%) developed TR. CK testing occurred in 78 (13.1%) patients with 29 (37.7%) of these having values followed until downtrending. AKI occurred in 63 (10.8%) patients within the entire study population. Among those with TR, nine (60%) patients developed AKI. Patients with TR had higher ISS (median 29 vs 18) and mortality (26.7% vs 8.9%).

**Discussion:**

Whilst TR appears rare without liberal screening, it is strongly associated with AKI. Given the poor outcomes, standardised monitoring, and liberal testing of CK could be justified in trauma patients with higher injury severity. This epidemiological data can help to define study populations and power future multicentre prospective studies on this infrequent yet morbid condition.

## Background

Rhabdomyolysis (RM) refers to a clinical syndrome caused by injury to striated muscles cells. The process is driven by energy homeostatic failure associated myocyte necrosis and/or loss of membrane integrity, which leads to exposure of cellular components into the blood stream [1, 2]. In particular, the release of myoglobin drives the major clinical complication of rhabdomyolysis, acute kidney injury (AKI) [3].

There are various aetiologies leading to RM with traumatic injury accounting for up to 29% of RM in intensive care unit (ICU) admitted patients [4)]. Among trauma patients, RM has been shown to be an independent risk factor for the development of AKI with the estimated incidence of this complication being found to be as high as 23% [5–8]. This highlights the importance of RM as AKI in trauma patients has demonstrated a 3.4–3.6 increase in relative risk for mortality [9, 10]. Prompt identification of this pathology therefore offers a unique opportunity, as RM is a potentially modifiable risk factor of AKI in trauma patients. Early aggressive management could offer a means of reducing the incidence of AKI and reducing mortality in these patients.

A commonly used laboratory test in the diagnosis of RM is the measurement of creatinine kinase (CK) concentration in the plasma. CK values allow for the diagnosis of RM whilst also having predictive capability for AKI in trauma patients [11–14]. This readily available test therefore plays an important role in the initial workup of the trauma patient, allowing risk stratification and initiation of early high-volume fluid therapy aimed at reducing AKI and thus patient mortality [15]. However, the at-risk population for RM remains poorly defined meaning clinicians have limited evidence-based tools to guide decision-making for testing.

Despite the recognised importance of RM as a clinical entity in trauma patients, there remains no evidence-based guidelines around screening, which CK value predicts complications, how best to monitor CK values, and at what value to begin therapy and best treatment strategies. Of the studies that do exist on RM in trauma patients, there are important limitations as many include only patients with CK values recorded during admission and investigate specific populations, leading to bias. The true incidence and natural history of this pathology therefore remains poorly understood.

We aim to describe the contemporary incidence, surveillance patterns, and treatment of TR at our level 1 trauma centre during a 12-month period. We hypothesise that current surveillance and treatment occurs in an ad hoc fashion allowing for an ability to improve patient care. Additionally, the results of this study will be used to design future prospective observational and interventional studies aimed at improving the characterisation, and investigating treatment options, in TR as a potentially modifiable predictor of AKI.

## Methods

### Definitions

#### AKI

The serum creatinine criteria of The Kidney Disease: Improving Global Outcomes (KDIGO) was used to define AKI staging in our cohort. Briefly, AKI 1 is characterised by an increase in serum creatinine of > 26.53 mol/L (0.3mg/dL) or 1.5–1.9 times increase above baseline; AKI 2 by 2.0–2.9 times increase above baseline; and AKI 3 by > 3 times increase above baseline, or an increase of > 353.68 mol/L (4.0mg/dL), or initiation of renal replacement therapy [16]. To improve specificity and clinical relevance, AKI was said to be present if patients met either stage 2 or stage 3 criteria at any stage during their admission [16, 17].

#### Baseline serum creatinine

Baseline serum creatinine was estimated using the Modification of Diet in Renal Disease equation by solving for serum creatinine and assuming a GFR of 100 [17]. Patients with a history of chronic kidney disease (CKD) were not assessed for AKI due to inability to accurately estimate baseline creatinine and prior need for renal replacement therapy.

#### Polytrauma

Polytrauma occurred when at least two AIS body regions were affected with the score above two in each (2 × AIS > 2 or ‘Newcastle’ definition of polytrauma) [18].

#### Rhabdomyolysis

RM was said to occur if CK levels exceeded 5000 international units (IU). Whilst variable CK values exist for RM, this value is commonly used within trauma literature [5, 6, 12, 19–21].

#### Multiple organ failure

It is defined as a DENVER score of 4 or greater after 48 h of ICU admission [22].

### Ethics

This study received ethics approval from the local health districts ethics committee (AU02212-02 and AU202303-14). It adheres to the provision of privacy and confidentiality of patient data and clinical information, including the State of New South Wales Health Records and Information Privacy Act 2002.

### Study design and participants

**Fig. 1:**
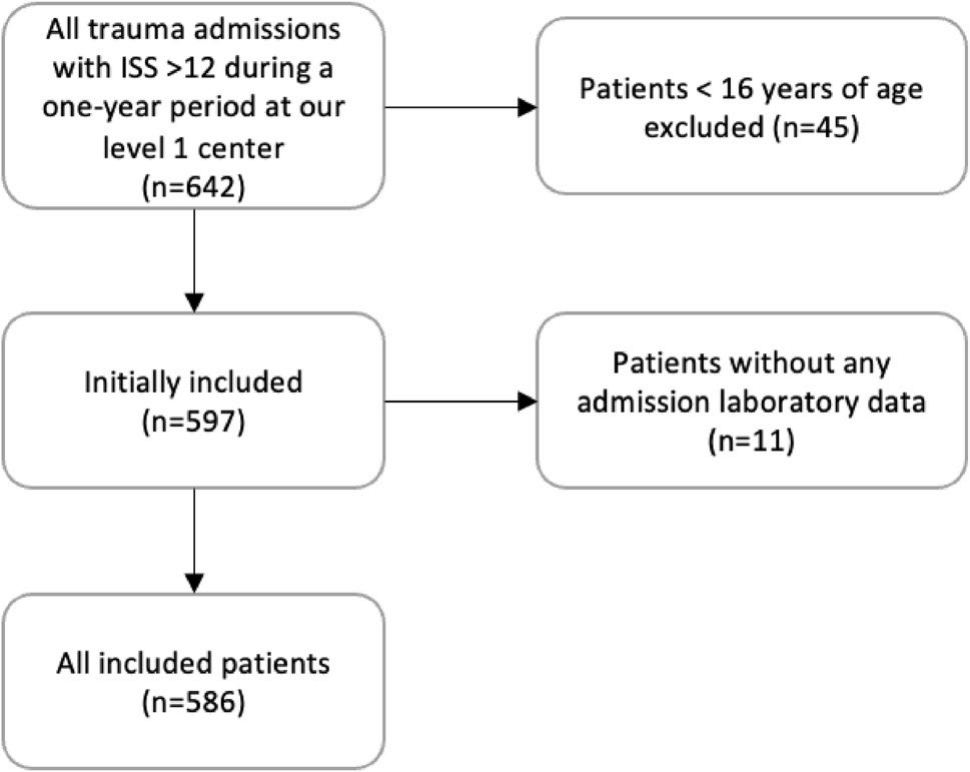
Flowchart of patient inclusion process

A 1-year retrospective study ending on December 31, 2019, was performed on all consecutive trauma admissions with an injury severity score (ISS) greater than 12 to our level 1 trauma centre. The year 2019 was chosen as no practice changes occurred during this period and it was unaffected by COVID-19. A single year duration was estimated to be adequate to provide the incidence figure for TR in our 1 million population region and serve as a multiplier for extrapolation to larger (state or country populations) populations for powering of future prospective observational and interventional studies. An ISS of greater than 12 was used to prevent underestimation of our primary outcome by excluding trauma patients with minimal injuries and with negligible risk of post-injury complications. Patients less than the age of 16 were excluded. Patients with no admission laboratory results or imaging study results were also excluded. All other patients were included for analysis. Figure 1 shows a flow diagram of the patient inclusion process. After exclusion of all patients less than 16 years of age, 597 patients had their medical records reviewed. Of these, 11 were excluded due to having no available laboratory data from their admission.

### Variables and data collection

Variables extracted from the Trauma Registry and focused chart review included demographics (age, sex), primary injury type (blunt vs penetrating), ISS, new injury severity score (NISS), pre-hospital mode of transport, extraction time and first ED vital signs (systolic blood pressure, heart rate, temperature, SpO2, GCS). Also collected was whether the patient was intubated, received paralytic agents, sedated, or required assisted respiration at the time of presentation to ED. Other variables collected included mortality, ICU length of stay (LOS), hospital LOS, ventilator days and need for renal replacement therapy (RRT).

Data extracted from patient records included administration of contrast at the time of presentation, administration of contrast during admission, quantity of contrast studies received and history of CKD.

Laboratory values extracted included admission CK, daily CK, admission serum creatinine, daily serum creatinine for the first 7 days of admission and highest creatinine value during admission to determine presence of AKI. The laboratory values for admission potassium, bicarbonate, corrected calcium, phosphate, lactate, base deficit (BD), haemoglobin (Hb), alanine transaminase (ALT) and aspartate transaminase (AST) were also collected.

Patient records were then reviewed for management received if diagnosis of rhabdomyolysis was confirmed. Variables collected relating to management included rate of fluid therapy, type of fluid, administration or mannitol or bicarbonate, and RRT.

### Study outcomes

The primary outcome of the study was the presence of TR defined as a CK level of greater than 5000 IU. Secondary outcomes included the development of AKI (KDIGO criteria), multiple organ failure (MOF), mortality, need for RRT, ICU length of stay (LOS) and hospital LOS.

### Statistical analysis

Incidence of TR was estimated as the proportion of the cohort who met the primary outcome. Descriptive statistics were used to summarise the characteristics, treatment and outcomes of patients with and without TR. Continuous variables were summarised as mean with standard deviation or median with inter-quartile range if the distribution was non-normal. Categorical variables were summarised as frequency count with percentage.

Among patients who underwent CK testing as surveillance for TR, patients with and without TR were compared descriptively and *p* values were reported from the *t* test for continuous variables (or the Wilcoxon–Mann–Whitney *U* test for variables with non-normal distributions) and the Chi-squared test for categorical variables.

## Results

### Whole cohort characteristics and outcomes

The mean age of the whole cohort (*n* = 586) was 53 years (22) and 424 (72%) were male. The median ISS was 18 (16, 25), 317 (54.2%) of patients met polytrauma criteria and 178 (30.4%) of all patients were admitted to the ICU. The mechanism of injury for 547 (93%) patients was blunt with the remainder (*n* = 39) being penetrating trauma. The median systolic blood pressure on arrival was 130mmHg (116, 146) and the median GCS was 15 (9, 15). All other admission vital signs and laboratory results for the cohort are found in Table 1. The median ICU LOS was 0 days (0, 1) with a median hospital LOS of 7 (3, 14). Median extraction time was 42.5 min (20, 60) and 395 patients arrived via ambulance (67.4%) and 134 (22.9%) via helicopter. Twelve patients (2%) developed MOF, 5 (0.9%) required RRT and 55 (9.4%) patients died.

**Table 1:** Characteristics and outcomes among patient populations

Characteristic	Whole cohort (*n* = 586)	CK tested (*n* = 78)	Non-rhabdomyolysis patients (*n* = 571)	Rhabdomyolysis patients (*n* = 15)
General characteristics				
Age (years)	53 (± 22)	50 (± 22)	54 (± 22)	35 (± 20)
Injury Severity Score	18 (16, 25)	25 (19, 38)	18 (16, 25)	29 (24, 42)
Blunt trauma	547 (93%)	72 (92.3%)	533 (93.3%)	14 (93%)
Penetrating trauma	39 (7%)	6 (7.7%)	38 (6.7%)	1 (7%)
Sex, male	424 (72%)	59 (76%)	410 (72%)	14 (93%)
CKD	19 (3.2%)	5 (6.5%)	18 (3.2%)	1 (7%)
Admitted to ICU	180 (30.7%)	61 (78.2%)	167 (29.2%)	13 (86.7%)
Polytrauma	317 (54.1%)	54 (70.1%)	306 (53.7%)	11 (73%)
Multiple organ failure	12 (2%)	6 (7.7%)	10 (1.8%)	2 (13.3%)
Hospital admission				
SaO2	98 (96, 99)	98.5 (96, 100)	98 (96, 99)	99 (97, 100)
Heart rate	84 (71, 99)	94 (78, 117)	84 (71, 98)	126 (113, 135)
Respiratory rate	18 (16, 20)	18 (16, 21)	18 (16, 20)	16 (14, 19)
Systolic blood pressure	130 (116, 146)	115 (98, 134)	130 (117, 148)	100 (90, 116)
GCS	15 (9, 15)	9.5 (3, 15)	15 (11, 15)	3 (3, 3)
Body temperature	36.3 (35.8, 36.8)	36.2 (35.3, 27)	36.3 (35.8, 36.8)	36.5 (35.0, 37.2)
Intubated	88 (15%)	29 (37.2%)	79 (13.8%)	9 (60%)
Paralytic agents	61 (10.4%)	21 (26.9%)	53 (9.3%)	8 (53.3%)
Sedated	69 (11.8%)	23 (29.5%)	61 (10.7%)	8 (53.3%)
Assisted respiration	101 (17.2%)	32 (41%)	92 (16.1%)	9 (60%)
Admission laboratory results				
CK		1200 (511, 3494)		5610 (2895, 15,268)
Creatinine	82 (71, 100)	103 (76, 138)	81 (70, 97)	124 (108, 150)
Potassium	4 (3.7, 4.3)	4.2 (3.7, 4.7)	4 (3.7, 4.3)	4.3 (4.1, 4.7)
Bicarbonate	23 (21, 25)	22 (18, 23)	23 (21, 26)	18 (17, 22)
Corrected calcium	2.33 (2.25, 2.45)	2.28 (2.21, 2.37)	2.34 (2.25, 2.45)	2.31 (2.25, 2.34)
Phosphate	1.09 (0.92, 1.31)	1.26 (1.02, 1.58)	1.07 (0.9, 1.30)	1.61 (1.56, 2.25)
Alanine transaminase	33 (21, 58)	46 (29, 105)	32 (21, 57)	84 (38, 265)
Aspartate transaminase	45 (31, 84)	84 (46, 165)	44 (31, 83)	164 (91, 312)
Lactate	2.2 (1.4, 3.3)	3.5 (2.1, 5.4)	2.1 (1.4, 3.2)	5.2 (3.8, 6.7)
Base excess	− 1.1 (− 3.6, 0.8)	− 4.2 (− 7.4, − 1.6)	− 0.9 (− 3.2, 0.9)	− 7.2 (− 8.6, − 6)
Haemoglobin	136 (125, 148)	133 (115, 144)	136 (125, 148)	139 (122, 147)
KDIGO				
AKI total	63 (10.8%)	35 (44.9%)	54 (9.5%)	9 (60%)
AKI grade 2	36 (6.1%)	20 (25.6%)	32 (5.6%)	4 (27%)
AKI grade 3	27 (4.6%)	10 (19.2%)	22 (3.9%)	5 (33%)
Outcomes				
CK > 5000 IU during admission	15 (2.6%)	15 (19.2%)		
CK > 1000 IU during admission	43 (7.3%)	43 (55.1%)		
ICU LOS	0 (0, 1)	2 (1, 9)	0 (0, 1)	2 (1, 10)
Hospital LOS (days)	7 (3, 14)	16 (6, 30)	7 (3, 13)	21 (9, 33)
Mortality	55 (9.4%)	14 (17.9%)	51 (8.9%)	4 (26.7%)
Ventilator days	0 (0, 0)	2 (0, 7)	0 (0, 0)	2 (1, 8)
Renal replacement therapy^a^	5 (0.9%)	3 (3.8%)	3 (0.5%)	2 (13.3%)
Prehospital mode of transport/extraction time				
Ambulance	395 (67.4%)	46 (59%)	390 (68.3%)	5 (33.3%)
Helicopter	134 (22.9%)	31 (39.7%)	124 (21.7%)	10 (66.7%)
Private vehicle	53 (9%)	1 (1.3%)	53 (9.3%)	0 (0%)
Other	3 (0.5%)	0 (0%)	1 (0.53%)	0 (0%)
Extraction time	42.5 (20, 60)	60 (40, 120)	40 (20, 60)	105 (80, 120)
Contrast studies				
Contrast administered within 24 h from presentation	329 (56.1%)	58 (74.3%)	318 (55.7%)	11 (70%)
Received contrast after 24 h from presentation	94 (16.0%)	25 (32%)	85 (14.9%)	9 (60%)
Number of contrast exposures during admission	1 (0, 1)	1 (1, 2)	1 (0, 1)	2 (1, 2.5)

Quantitative variables are summarized as either mean (standard deviation) or median (quartile 1, quartile 3). Categorical variables are summarized as frequency count and percentage

^a^All cases of renal replacement therapy were CRRT

### Primary outcome: incidence of rhabdomyolysis and CK testing

There was a total of 15 (2.56%) patients with laboratory results consistent with the study’s definition of TR. Among the entire cohort, 78 (13.1%) patients had CK tested during their admission.

Characteristics and outcomes of patients with traumatic rhabdomyolysis:

Patients with TR had a mean age of 35 (20), 14 (93%) were male and 13 (86.7%) were admitted to the ICU. The median ISS was 29 (24, 42), 11 (73%) patients met polytrauma criteria and 14 (93%) had a blunt mechanism of injury. The median systolic blood pressure was 100mmHg (90, 116) and the median GCS was 3 (3, 3). At admission, nine (60%) were intubated and were requiring assisted ventilation. Patients with TR had a median extraction time of 105 min (80, 120). Ten (66.7%) arrived via helicopter with the rest arriving via ambulance (*n* = 5). The median ICU LOS, hospital LOS and ventilator days were 2 (1, 10), 21 (9, 33) and 2 (1, 8), respectively. Two patients (13.3%) developed MOF, two (13.3%) required RRT and four (26.7%) of the patients with rhabdomyolysis died.

### Treatment among those with traumatic rhabdomyolysis

Of the patients with TR, nine (60%) were treated with high-volume fluid therapy with rates exceeding daily output and/or maintenance requirements. Among those who received high-volume fluid therapy, two (13.3%) received RRT, two (13.3%) received sodium bicarbonate and two (13.3%) received mannitol. All patients receiving mannitol received this for traumatic brain injury and not as a part of their management for TR. Types of crystalloid therapy used included normal saline (*n* = 4), Hartmann’s solution (*n* = 3) and a combination of both solutions (*n* = 8). In the instances of RRT, continuous renal replacement therapy (CRRT) was used in all cases.

### Incidence of AKI among patient populations

The overall incidence of AKI among trauma patients was 10.8% (*n* = 63). Of these, 36 (6.1%) met stage 2 criteria and 27 (4.6%) met stage 3 criteria. The overall incidence of AKI among those without TR was 9.5% (*n* = 54). Among those with TR, nine (60%) patients developed either stage 2 or stage 3 AKI. Of these, four (27%) met stage 2 criteria and five (33%) met stage 3 criteria. Among patients with TR who received high-volume fluid therapy, four (44.4%) developed AKI. Among the six patients who did not receive high-volume fluid therapy, five developed AKI (83%).

### Surveillance of CK levels: characteristics of those being investigated for traumatic rhabdomyolysis

Seventy-eight (13.1%) patients had CK levels tested during their admission. These patients had a mean age of 50 years (20), 59 (76%) were male and the median ISS was 25 (19, 38). Fifty-four (70.1%) of those with their CK values tested met polytrauma criteria. Seventy-two (92.3%) had a blunt mechanism of injury. The median extraction time was 60 min (40, 120), 46 (59%) arrived via ambulance and 31 (39.7%) arrived via helicopter. Regarding ICU-admitted trauma patients, 61 of the 180 patients (33.9%) had their CK levels tested during admission. The remainder of the admission laboratory results, and vital signs for patients who were tested for CK are found in Table 1. The median admission CK value was 1200 (511, 3494) and the mean time of the first CK taken was within 24 h from presentation.

## Discussion

This study was a retrospective cohort study at our level 1 trauma centre aimed at investigating the incidence, current monitoring and management of TR. We found an overall incidence for TR of 2.6% for all trauma patients with an ISS greater than 12, and for ICU-admitted trauma patients an incidence of 7.2%. This result is at risk of underestimation as only 13.1% of patients had CK values tested. The incidence of TR varies within the literature depending on definition and population studied. Incidences have been shown to be as high as 31% in combat environments [5]. Among civilian studies, in ICU-admitted trauma patients, an incidence between 11% and 13.2% was found [6, 13, 21].

TR within our study was associated with a higher ISS, worse physiological derangement at the time of admission, reduced GCS, younger age and helicopter transport. Patients with TR had a similar incidence of blunt and penetrating trauma as the general trauma cohort. The incidence of polytrauma was higher among TR patients; however, not all TR patients met polytrauma criteria and two patients had isolated head injuries. These results demonstrate heterogeneity among trauma patients who develop RM. Classically, patients with large trauma burden, crush injuries involving the extremities and those with longer extraction times have been considered at highest risk of developing TR [23]. Other important risk factors have emerged within the literature including gunshot wounds, vascular injuries, abdominal injuries, older age and a body mass index greater than 30 kg/m^2^ well [6, 15, 24]. Due to this heterogeneity and the absence of evidence-based risk stratification tools, clinicians must have a high index of suspicion for RM within all trauma patients and thus liberal screening is essential.

The diagnosis and risk stratification in RM relies upon a variety of clinical and biochemical findings. One important aspect of this initial workup is the quantitative assessment of CK levels in serum. This marker commonly provides the foundation for the diagnosis of RM and is a readily available investigation within most trauma centre settings [25]. Beyond the diagnostic yield of this test, within trauma cohorts, raised CK values correspond to the risk of developing AKI and inpatient mortality [11–14, 26–28]. The CK value associated with acute kidney injury varies between 1695 IU and 14,494 IU with the majority of articles quoting a value between 3000 and 5000IU [2–14, 26, 27]. Various other laboratory findings such as raised aminotransferases, venous bicarbonate and deranged electrolytes have also shown an association with poor outcomes; however, no formalised prediction tool has been validated within a prospective cohort methodology specifically for determining the risk of poor renal outcomes in trauma patients [29–31]. Urinary myoglobin is another commonly utilised means of evaluating the presence and extent of RM. There exists conflicting literature around its yield with some studies stating it to be a weak predictor of AKI and others concluding increased sensitivity when compared to CK [14, 20, 32]. Due to the lack of urinary myoglobin monitoring performed at our centre, we were unable to comment on its utility and use in the diagnosis of TR.

Just as there is no prediction tool for deciding who is at risk of acute renal failure and mortality in TR, there is also no formal guideline on the screening and monitoring of CK values. The recent consensus document by the American Association for the Surgery of Trauma (AAST) suggests CK values be a part of the initial workup for patients deemed at risk [15]. From this recommendation, the difficulty is in consistent determination of who is ‘at risk’. The AAST also suggest serial CK measurement with values being followed until the peak concentration is identified [15]. This peak can occur up to 5 days from the initial insult with it usually occurring between 24 and 72 h [15, 25]. Among ICU-admitted trauma patients, only 33.9% had CK concentrations evaluated during their admission. Whilst not all trauma admissions will have sufficient risk factors for the development of TR, patients requiring ICU admission are likely to have high injury severity and physiological derangement to justify investigation [3]. Among those who had CK measurements, only 37.7% had further tests followed until downtrending, preventing accurate diagnosis, decision-making and treatment [15].

TR has been identified as an independent risk factor in the development of AKI [7, 8, 33]. The incidence of AKI among TR patients varies ranging between 12.2% and 23.5% [5, 12]. Our study found an incidence of clinically relevant AKI of 60% with 13.3% of patients requiring RRT. AKI in trauma cohorts is a challenging clinical entity responsible for increased mortality and high resource use within the ICU [9, 10, 34]. Despite this, the majority of the risk factors for AKI in trauma patients are non-modifiable, including injury severity and degree of physiological derangement [10]. Many of the modifiable risk factors in AKI are yet to be determined in high-quality prospective studies and with previously feared aspects of care such as intravenous contrast administration recently showing no association with AKI [35]. Therefore, in TR-associated AKI, clinicians have a unique opportunity to implement early treatment to reduce the burden and incidence of this morbid and lethal complication. This is further suggested by our results demonstrating a higher incidence of AKI in those with TR who did not receive high-volume fluid therapy.

The management of TR is varied, as there is a paucity of literature to guide practice. Various strategies have been previously implemented including high-volume fluid therapy, sodium bicarbonate, mannitol and early use of RRT. The recommended treatment for RM is the use of high-volume fluid therapy with either saline or a solution such as Lactated Ringer’s, with other therapies lacking evidence [15, 36]. Initial high-volume fluid therapy has been found to be otherwise detrimental during early care of polytrauma patients and therefore accurate and timely identification of patients at risk of TR is crucial to tailored patient care [37]. Within our study, we found both Lactated Ringer’s solution and saline solution being used for patients. Only 60% of patients with TR had evidence of high-volume fluid therapy.

With the current gap in literature and rare incidence of TR, future prospective studies will require multicentre collaboration to achieve adequate sample sizes capable of demonstrating statistically significant results. We aimed to provide data on the contemporary incidence of TR to assist with powering these much-needed prospective trials. The 15 patient per year from a 1 million person region allows us the calculation of 1.5/100,000/year incidence and enables the design of prospective observational studies to address the unanswered question in a multicentre fashion. Our state within Australia has seven major trauma centres and with the population-based extrapolation we can expect 100–110 cases enrolled prospectively. This is a reasonable power for prospective, even interventional studies, which we can power and calculate effect sizes based on our 12-month single-centre study. Due to the inherent limitations of retrospective studies, we were unable to demonstrate causation regarding risk factors for the development of TR. However, we were able to indicate which patients could be perceived as high risk and thus be considered for inclusion in future trials. Therefore, future studies can be guided by our results and patient characteristics to assist with early identification of TR patients (Table 2).

**Table 2:** Characteristics and outcomes among patients either with or without laboratory evidence of RM who had CK tested during admission

Characteristic	Rhabdomyolysis patients (*n* = 15)	Non-rhabdomyolysis (with CK tested) (*n* = 63)	*p* value
General characteristics			
Age (years)	35 (± 20)	54 (± 20)	0.0019
Injury Severity Score	29 (24, 42)	25 (17, 33)	0.0525
New Injury Severity Score	42 (27, 54)	27 (22, 43)	0.9800
Blunt trauma	14 (93%)	58 (92%)	0.8683
Penetrating trauma	1 (7%)	5 (7.9%)	0.8683
Sex, male	14 (93%)	45 (71%)	0.0757
CKD	1 (7%)	4 (6.3%)	0.9640
Admitted to ICU	13 (86.7%)	48 (76%)	0.3771
Hospital admission			
SaO2	96.3 (± 5.4)	96.5 (± 8.1)	0.9495
Heart rate	117.9 (± 28)	92.1 (± 23.4)	0.0010
Respiratory rate	16.1 (± 4.2)	19.3 (± 5.4)	0.0464
Systolic blood pressure	102.9 (± 25.8)	119.5 (± 27.8)	0.0525
GCS	4.9 (± 5.1)	9.3 (± 5.9)	0.0097
Body temperature	36.2 (± 1.7)	36 (± 1.8)	0.6244
Intubated	9 (60%)	20 (71%)	0.2357
Paralytic agents	8 (53.3%)	13 (54%)	0.0648
Assisted respiration	9 (60%)	23 (82%)	0.5586
Admission laboratory results			
Creatinine	134.6 (± 46.4)	105.3 (± 44.7)	0.0264
Potassium	4.5 (± 0.9)	4.2 (± 0.8)	0.2736
Corrected calcium	2.3 (± 0.1)	2.3 (± 0.2)	0.4796
Phosphate	1.7 (± 0.7)	1.4 (± 0.7)	0.1299
Alanine transaminase	233.5 (± 307.7)	83 (± 131)	0.0052
Aspartate transaminase	441.8 (± 828.1)	125.9 (± 157.8)	0.0076
Base excess	− 7.5 (± 3.7)	− 4.4 (± 5.7)	0.0525
Haemoglobin	134.8 (± 26.1)	124.1 (± 28.8)	0.1908
Outcomes			
ICU LOS	2 (1, 10)	2 (0, 9)	0.7110
Hospital LOS (days)	21 (9, 33)	14 (6, 28)	0.5739
Mortality	4 (26.7%)	10 (16%)	0.3276
Ventilator days	2 (1, 8)	1 (0, 7)	0.3722
Prehospital mode of transport/extraction time			
Ambulance	5 (33.3%)	41 (65%)	0.0580
Helicopter	10 (66.7%)	21 (33%)	0.0580
Private vehicle	0 (0%)	1 (1.6%)	0.0580
Other	0 (0%)	0 (0%)	0.0580

Quantitative variables are summarized as either mean (standard deviation) or median (quartile 1, quartile 3). Categorical variables are summarized as frequency count and percentage

## Limitations

There are several important limitations to our study. Firstly, the methodology of our study was a retrospective cohort study which has several inherent limitations including selection bias and risk of confounding. Furthermore, as CK values were not tested in all patients, we do not know the true incidence of TR within our cohort. The study has low numbers of patients with our outcome of RM and therefore we were unable to complete robust statistical analysis. We assumed baseline creatinine values to determine the presence of AKI and results are therefore at risk of bias.

## Conclusion

Without liberal screening, TR appears to be a rare phenomenon among our predominantly blunt trauma cohort, but almost uniformly associated with AKI and with a large proportion requiring RRT. A standardised approach in monitoring is required to reduce the incidence of AKI and mortality among critically injured trauma patients. This study can assist with the design of future prospective multicentre studies aimed at improving characterisation of TR and guiding treatment options.

## Data Availability

The data that support the findings of this study are available on request from the corresponding author.
